# Spontaneous and induced abortions in women with a diagnosis of gestational related neoplasm: a population-based linkage study in Lombardy, 2010–2020

**DOI:** 10.1186/s12905-023-02685-6

**Published:** 2023-11-08

**Authors:** Giovanna Esposito, Matteo Franchi, Claudia Santucci, Giovanna Scarfone, Fabio Parazzini, Carlo La Vecchia, Giovanni Corrao, Eva Negri

**Affiliations:** 1 Department of Clinical Sciences and Community Health, University of Milan, Milan, Italy; 2 National Centre for Healthcare Research and Pharmacoepidemiology, Milan, Italy; 3 Department of Statistics and Quantitative Methods, Unit of Biostatistics, Epidemiology and Public Health, University of Milano-Bicocca, Milan, Italy; 4 Department of Obstetrics, Gynecology and Neonatology, University of Milan, Fondazione IRCCS Ca’ Granda Ospedale Maggiore Policlinico, Milan, 20122 Italy; 5 Department of Medical and Surgical Sciences, University of Bologna, Bologna, Italy

**Keywords:** Pregnancy associated cancer, Abortion, Pregnancy outcome, Miscarriage

## Abstract

**Background:**

A diagnosis of cancer during pregnancy or within one year after the end of pregnancy is a major clinical and public health issue. The current study aimed at estimating the incidence of pregnancy-associated cancer (PAC) and assessing whether the risk of abortion is increased in women diagnosed with cancer.

**Methods:**

This population-based cohort study used the regional healthcare utilization (HCU) databases of Lombardy, the largest region in Italy, to identify the women who delivered between 2010 and 2020. PAC were identified by oncological ICD-9-CM codes reported in the hospital discharge forms. We computed the ratio of PAC cases to the total number of pregnancies. Following a diagnosis of PAC, the prevalence ratio (PR) of abortion and the corresponding 95% confidence interval (CI), was estimated using a log-binomial model adjusted for maternal age.

**Results:**

During the study period, 926 women who gave birth (1.29 cases per 1000 births) and 341 women who had an abortion (1.52 cases per 1000 abortions) were diagnosed with PAC. Regardless of the outcome of pregnancy, the risk of PAC increased with increasing age. The rate of PAC was initially lower among births, but it came very close to the rate of PAC among abortions in the last two calendar years. The proportion of abortions among women with PAC gradually decreased from 27.7% in 2010–2012 to 18.5% in 2019–2020 (*p*-value < 0.001). Overall, a diagnosis of PAC was related to an approximately 10% increased risk of abortion (PR = 1.11, 95%CI:1.01–1.22). However, no association was observed in 2019–2020 (PR = 0.87, 95%CI:0.65–1.17). Considering only diagnoses made during the first trimester of pregnancy, the risk of abortion was about 2.5 times higher (PR = 2.53, 95%CI:2.05–3.11) and the risk of induced abortion was almost 4 times higher (PR = 3.71, 95%CI:2.82–4.90).

**Conclusion:**

In this population the risk of abortion was about 10% higher in women with PAC than in women without PAC. However, this association tended to decrease in more recent calendar periods. This trend seemed to be influenced more by spontaneous than by induced abortions.

**Supplementary Information:**

The online version contains supplementary material available at 10.1186/s12905-023-02685-6.

## Background

The diagnosis of a neoplasia during pregnancy or in the year following childbirth or abortion, i.e. pregnancy-associated cancer (PAC), is a major challenge requiring a multidisciplinary approach to optimize the management of both cancer and pregnancy, and a public health issue due to the increasing maternal age over recent calendar years and the consequent higher cancer incidence. A recent systematic review [1] of population-based studies reported an overall incidence of PAC of approximately 1 case in 1000 pregnancies.

According to the clinical practice guidelines of the European Society for Medical Oncology, although systemic treatment with chemotherapy is associated with a high risk of miscarriage and possibly congenital malformations, most cancer treatments during pregnancy are possible [2]. There is therefore no definite reason to interrupt pregnancy following a diagnosis of cancer in order to start chemotherapy, unless the severity of the oncological pathology is life-threatening. A diagnosis of cancer may influence not only miscarriage but also the decision to terminate the pregnancy because of its social and psychological implications, irrespective of the clinical condition.

The impact of PAC on pregnancy outcome (i.e. birth or abortion) has been inadequately investigated and the results remain controversial. In two population-based studies from different areas of Italy, abortion was more frequent in women with PAC [3, 4]. In contrast, in another Italian cohort study, the risk of PAC was lower in pregnancies ending in abortion (1.25 per 1000) than in those ending in birth (1.35 per 1000) [5]. A Danish study, based on national registries and conducted between 1977 and 2006, found that almost one third of women diagnosed with PAC had an abortion, and of these, more than 65% were induced [6].

Comprehensive monitoring of the temporal trends of PAC is paramount in reproductive health to quantify the magnitude of the event and to understand the changing landscape of relation between oncology and pregnancy. Investigation of specific issues is needed to identify areas where additional awareness is needed to improve the management of PAC.

The current study provides evidence on the incidence and trends of PAC in births and abortions in Lombardy, northern Italy, during the period 2010–2020.

## Material and methods

### Data sources and study cohort

The data were obtained from the regional healthcare utilization (HCU) databases of Lombardy, the largest region in Italy, accounting for about 17% of the national population with about 10 million inhabitants. In Italy, the entire resident population benefits from healthcare services provided by the National Health Service (NHS). In Lombardy, the NHS has been linked to an automated system of databases since 1997. These include: (i) an archive of NHS beneficiaries (residents who receive NHS assistance) in the region, with socio-demographic and administrative information; (ii) hospital discharge data [scheda di dimissione ospedaliera (SDO)], reporting diagnoses and procedures of hospital inpatients [coded according to the International Classification of Diseases, Ninth Revision, Clinical Modification (ICD-CM-9)], hospitalisation-related costs [coded according to the national diagnosis related group (DRG) system] and other detailed information on hospitalisation; and (iii) the certificates of birth assistance [certificato di assistenza al parto (CedAP)] registry, which provides detailed information on the parents’ socio-economic status, the course of pregnancy and birth, and the health of the newborn. In addition, chemotherapies administered in the inpatients setting or directly administered in the outpatient setting and day-hospital coded according to the Anatomical Therapeutic Chemical (ATC) classification system were also available in the HCU databases. For each woman, we linked these databases via a unique identification code, which was automatically anonymised in full respect of individual’s privacy. As a result, no ethics committee approval is required to analyse these data.

We identified all the pregnancies that occurred in the Lombardy region between 1^st^ January 2010 and 31^st^ December 2020 in women who were NHS beneficiaries. We selected all SDO with DRG codes related to births and abortions (i.e., 370–375 and 380–381, respectively) and verified that each SDO reported diagnosis or procedure codes related to birth or abortion. Exclusion criteria were as follows: (i) pregnancies of women not resident in Lombardy (at least since three years before the estimated conception date and one year after the birth date), (ii) pregnancies of women aged less than 15 or more than 55, (iii) pregnancies of women with a diagnosis of cancer in the five years before the conception, and only for births (iv) those occurred before the 22nd week or after the 42nd week of pregnancy.

### Definition of abortion

According to the World Health Organization (WHO), abortion is defined as the termination of pregnancy before 22 weeks’ gestation. We identified all spontaneous (ICD-9-CM codes: 632. and 634.) or induced abortions (ICD-9-CM code: 635.) that occurred to inpatient women, both those without dilation and curettage (DRG: 380) and those with dilation and curettage, aspiration or hysterotomy (DRG: 381). Fetal death later in pregnancy, i.e. stillbirth, was considered a birth.

### Definition of PAC

Information on cancer diagnosis was obtained from the SDO database by selecting, for women in our cohort, all SDO reporting an ICD-9-CM code related to neoplasm, i.e., a diagnosis of cancer in the principal or secondary diagnoses. Women with PAC were defined as having at least one SDO with an ICD-9-CM code for malignant cancer (diagnostic codes:140.-208.), either as a principal or secondary diagnosis, during the period from conception to birth or during the following 12 months. In the case of abortions, it was not possible to determine the date of conception. Therefore, we could not determine the gestational age at the time of the abortion and, consequently, the time of cancer diagnosis according to the trimester of pregnancy. However, most abortions were expected to occur in the first trimester, and only a few within 180 days of pregnancy (under Italian law 194 on abortion). Thus, among women who had an abortion, those with PAC were defined as having an ICD-9-CM code for cancer, either in the principal or in the secondary diagnoses, in the period between conventionally three months before and 12 months after the abortion. Women were excluded if the cancer was reported as a secondary diagnosis and the primary diagnosis was not related to cancer or pregnancy. We considered the admission date of the first SDO reporting a malignancy diagnosis as the date of cancer diagnosis and classified cancers according to the time of diagnosis: during or after pregnancy.

In addition, the frequency of chemotherapy in women diagnosed with PAC was considered according to pregnancy outcome, and the pattern of treatment was described according to whether it was started during pregnancy, in the first six months, or in the late six months after the end of pregnancy. A woman was considered to be receiving chemotherapy if she had at least one prescription on File F registry for chemotherapy (ATC code: L01), if she reported a outpatient specialist service that refers to access to day hospital for chemotherapic therapies (codes: MAC01, MAC02, MAC03, MC04, 9925), or if she had at least a SDO related to chemotherapic treatment (diagnostic code: V581, intervention codes: 9925, 9928).

### Data analyses

We calculated the rate of PAC occurrence per 1000 pregnancies. We calculated the rate of PAC in strata of pregnancy outcome (i.e., birth or abortion) and the trend over calendar years, i.e., the year in which the birth or abortion occurred. The Cochran-Armitage test for trend was used to assess a linear trend in the PAC rate over the period considered. The Chi-square test was used to test for differences in pregnancy outcomes between PAC and cancer-free women over the period considered.

Moreover, the effect of PAC on the risk of abortion, overall and considering only induced abortion, was estimated using a log-binomial model and providing prevalence ratios (PR) and the corresponding 95% confidence intervals (CI) adjusted for maternal age.

These analyses were repeated in the subset of PAC diagnosed in the first trimester of pregnancy and in the subset of PAC diagnosed in the second or third trimester or in the year after the end of pregnancy. We also evaluated the distribution of cases according by time of diagnosis (during pregnancy: first, second and third trimester; post-pregnancy: 0–6 months, 7–12 months).

All analyses were performed using the Statistical Analysis System Software (version 9.4; SAS Institute, Cary, NC, USA).

## Results

### Study population

During the study period 2010–2020, 1,166,778 pregnancies were identified. Out of these, 896,256 ended with birth and 270,522 with an abortion. After the exclusion of SDO related to birth without a matching CedAP form, births or abortions of women not resident in Lombardy or aged less than 15 or more than 55 years, births before the 22nd week or after the 42nd week, and births or abortions of women with a diagnosis of cancer in the five years before the conception, we obtained a cohort of 941,395 pregnancies (717,106 births and 224,289 abortions) (see Supplementary material—Figure S1).

### Incidence of PAC and temporal trends

**Fig. 1:**
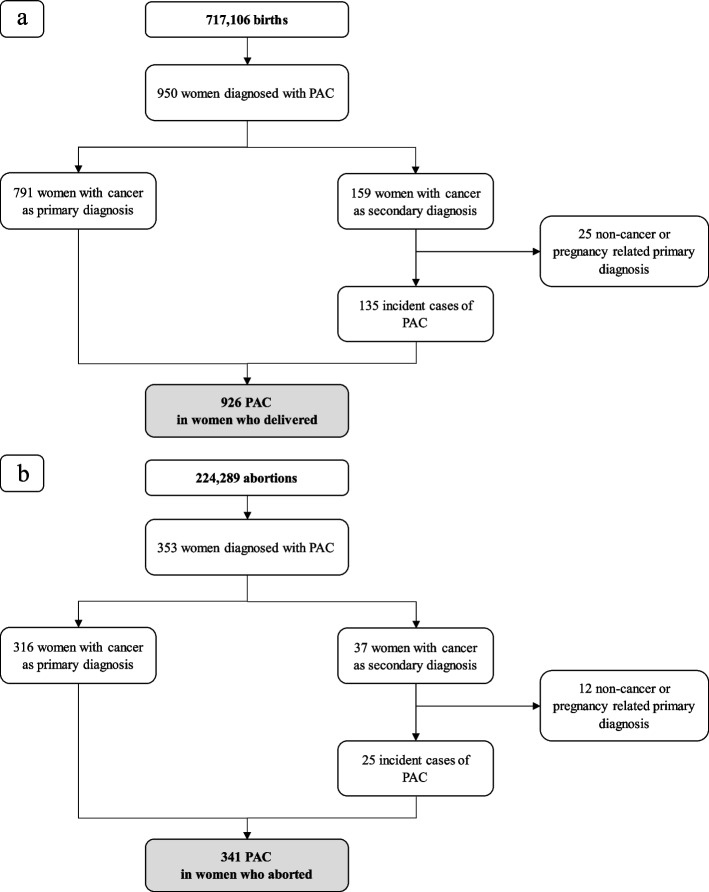
Flow-chart of study cohort of births (**a**) and abortions (**b**). Lombardy Region, 2010–2020

A total of 1267 cases of PAC were identified, corresponding to 1.35 per 1000 pregnancies. Of these, 926 (73.1%) women gave birth (1.29 cases per 1000 births), and 341 (26.92%) women had an abortion (1.52 cases per 1000 abortions) (Fig. 1). Among women with PAC who gave birth, 181 (19.5%) were diagnosed during pregnancy and 745 (80.5%) were diagnosed in the year after the birth. In the subgroup of women with PAC who had an abortion, 34 (10.0%) were diagnosed in the three months before the hospital admission for abortion and 307 (90.0%) were diagnosed in the year after the interruption of pregnancy.

Among cancers diagnosed in the year after the end of the pregnancy, diagnoses during the first 6 months were more frequent in women who aborted in comparison with women who delivered (*p*-value = 0.01) (see Supplementary material-Table S1).

The most common cancer sites were breast (30.1% for births and 37.4% for abortions), thyroid (19.4% for births and 20.3% for abortions), lymphomas (10.3% for births), and cervix cancer (7.9% for abortions). In addition, choriocarcinoma was more frequent in women who aborted than in women who gave birth (4.4% versus 0.6%, respectively).

Table 1 shows the distribution of PAC and cancer-free women and the rate of PAC in strata of pregnancy outcome according to maternal age and calendar year. Only 10.7% of women diagnosed with PAC were under 30 years. The risk of PAC was 0.59 per 1000 pregnancies in women aged less than 30 years and increased with advancing age, reaching 2.62 in women aged over 40. In women aged over 35, the rate of PAC was higher births for abortions than for births; in women aged 35–40, the rate was 2.01 per 1000 for abortions and 1.69 per 1000 for births, and in women aged over 40 years, the rates were 3.06 and 2.35, respectively. There was no significant difference in the distribution of PAC by calendar year.

**Table 1:** Distribution of pregnancy-associated cancer (PAC) and cancer-free women and the rate of PAC in strata of outcome of pregnancy according to maternal age and calendar year. Lombardy Region, 2010–2020

	**Births**			**Abortions**		
	**PAC women** **N (%)**	**Cancer-free** **women** **N (%)**	**Rate of PAC per 1000 pregnancies** **(95%CI)**	**PAC women** **N (%)**	**Cancer-free** **women** **N (%)**	**Rate of PAC per 1000 pregnancies** **(95%CI)**
*Maternal age*						
< 30	98 (10.6)	163,677 (22.9)	0.60 (0.50–0.70)	38 (11.1)	66,540 (29.7)	0.57 (0.42–0.78)
30–34	281 (30.4)	250,098 (34.9)	1.12 (1.10–1.26)	56 (16.4)	51,695 (23.1)	1.08 (0.83–1.40)
35–40	425 (45.9)	250,557 (35.0)	1.69 (1.54–1.86)	148 (43.4)	73,447 (32.8)	2.01 (1.71–2.36)
> 40	122 (13.2)	51,848 (7.2)	2.35 (1.97–2.80)	99 (29.0)	32,266 (14.4)	3.06 (2.51–3.72)
*Calendar year at pregnancy*						
2010	97 (10.5)	68,400 (9.6)	1.42 (1.16–1.73)	39 (11.4)	24,565 (11.1)	1.59 (1.16–2.17)
2011	99 (10.7)	72,050 (10.1)	1.37 (1.13–1.67)	34 (10.0)	24,331 (10.9)	1.40 (1.00–1.95)
2012	80 (8.6)	71,350 (10.0)	1.12 (0.90–1.39)	35 (10.3)	23,741 (10.6)	1.47 (1.06–2.05)
2013	78 (8.4)	69,039 (9.6)	1.13 (0.90–1.41)	34 (10.0)	23,269 (10.4)	1.46 (1.04–2.04)
2014	88 (9.5)	68,646 (9.6)	1.28 (1.04–1.58)	36 (10.6)	22,527 (10.1)	1.60 (1.15–2.21)
2015	82 (8.9)	67,153 (9.4)	1.22 (0.98–1.51)	38 (11.1)	20,810 (9.3)	1.82 (1.33–2.50)
2016	81 (8.8)	65,184 (9.10)	1.24 (1.00–1.54)	34 (10.0)	19,754 (8.8)	1.72 (1.23–2.40)
2017	82 (8.9)	62,975 (8.8)	1.30 (1.05–1.61)	31 (9.1)	18,347 (8.2)	1.69 (1.19–2.39)
2018	80 (8.6)	60,172 (8.4)	1.33 (1.07–1.65)	24 (7.0)	16,973 (7.6)	1.41 (0.95–2.10)
2019	85 (9.2)	57,541 (8.0)	1.48 (1.19–1.82)	16 (4.7)	15,880 (7.1)	1.01 (0.62–1.63)
2020	74 (8.0)	53,670 (7.5)	1.38 (1.10–1.73)	20 (5.9)	13,751 (6.1)	1.45 (0.94–2.24)

*CI* Confidence interval

**Fig. 2:**
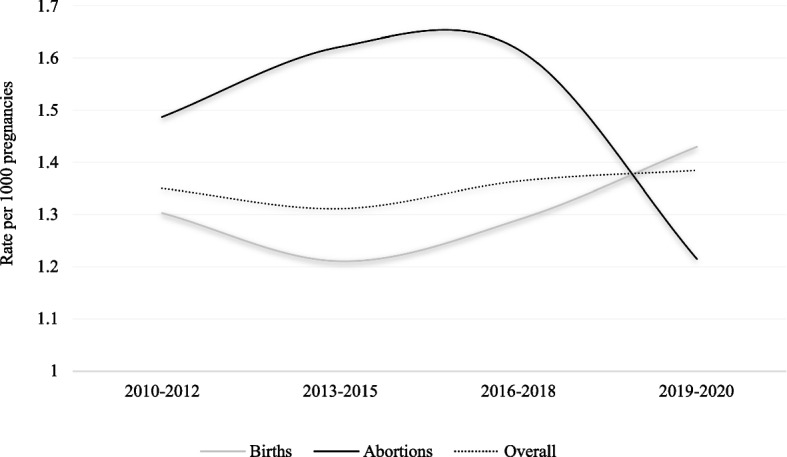
Temporal trends of rate (per 1000 pregnancies) of pregnancy associated cancers (PAC) in the cohort of births and in the cohort of abortions. Lombardy Region, 2010–2020

Figure 2 shows the time trends of PAC rates for births and abortions from 2010 to 2020. No trend was observed, but a decrease was observed in the subgroup of women who had an abortion during the period 2019–2020.

Chemotherapy was administered to 44.0% of women diagnosed with PAC who gave birth and to 47.2% of women who aborted. The patterns of treatment are given in Supplementary material (Table S2). 

### Risk of abortion among PAC women

**Fig. 3:**
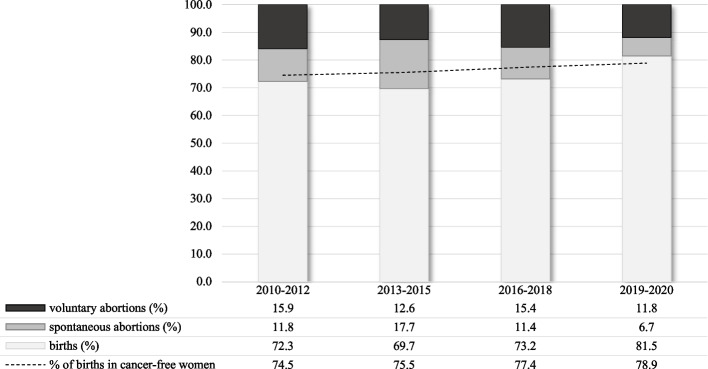
Outcomes of pregnancy in the cohort of women diagnosed with pregnancy associated cancers (PAC). Lombardy Region, 2010–2020

Figure 3 shows the distribution of pregnancy outcomes among women with PAC by calendar year. The proportion of abortions among women diagnosed with PAC decreased progressively over the study period from 27.7% in the period 2010–2012 to 18.5% in the period 2019–2020 (*p*-value < 0.001). Over the entire study period, a diagnosis of PAC was associated with an approximately 10% increased risk of abortion (PR = 1.11, 95%CI: 1.01–1.22). If we only considered PAC diagnosed in the first trimester of pregnancy, we identified 55 cases and observed a high proportion of abortions (PR = 2.53, 95%CI: 2.05–3.11), especially induced abortions (PR = 3.71, 95%CI: 2.82–4.90). However, when only PAC diagnosed after the first trimester or in the year after the end of pregnancy were considered, no association emerged (PR = 1.04, 95%CI: 0.95–1.15).

Table 2 provides PR of abortion in women diagnosed with PAC at three-years intervals. In the first years of the period considered, a positive association between PAC and abortion emerged, although of borderline significance due to the small number of cases; the PR was 1.08 (95%CI: 0.92–1.26) in 2010–2012, 1.21 (95%CI: 1.03–1.42) in 2013–2015 and 1.17 (95%CI: 0.98–1.40) in 2016–2018. In contrast, no association was observed in 2019–2020 (PR = 0.87, 95%CI: 0.65–1.17). In the subset of PAC diagnosed during the first trimester of pregnancy, the association between PAC and abortion was observed in every time interval considered.

**Table 2:** Prevalence ratios (PR) and related 95% confidence intervals (CI) of abortion in women diagnosed with pregnancy associated cancer (PAC). Lombardy Region, 2010–2020

			**PR**^**a**^** (95%CI)**		
	**Cancer-free women,** **N (%)**	**PAC women,** **N (%)**	**All the diagnoses**	**Diagnoses during the first trimester**	**Diagnoses during the second trimester or later**
**2010–2012**	72,637 (25.5)	108 (28.1)	1.08 (0.92–1.26)	2.77 (2.08–3.69)	0.99 (0.84–1.18)
**2013–2015**	66,606 (24.5)	108 (30.3)	1.21 (1.03–1.42)	1.99 (1.18–3.36)	1.18 (1.00–1.39)
**2016–2018**	55,074 (22.6)	89 (26.8)	1.17 (0.98–1.40)	2.68 (1.83–3.91)	1.09 (0.90–1.32)
**2019–2020**	29,631 (21.0)	36 (18.5)	0.87 (0.65–1.17)	2.67 (1.41–5.07)	0.80 (0.59–1.10)

^a^Estimated from log-binomial regression model adjusted for maternal age

## Discussion

The present study provides a comprehensive insight into the incidence and trends of PAC in women who gave birth and in women who had an abortion. The study of trends in PAC in relation to pregnancy outcomes allows us to make inferences on improvement of the management of oncological conditions during pregnancy and the potential coexistence of reproduction and cancer. In our cohort, the PAC rate was higher in women who had an abortion than in women who gave birth. The risk of abortion was about 10% higher in women with PAC than in women without PAC. However, the differences tended to decrease in more recent calendar periods. This tendency seemed to be influenced more by spontaneous abortions than by induced ones.

The association between abortion and PAC has rarely been studied. In an investigation conducted among Italian women from Piedmont, Veneto, Tuscany, and Apulia, the risk of PAC per 1000 abortions was lower than the risk per 1000 births (1.25 vs 1.35, respectively) [5]. However, in a study conducted in Lombardy in 2012, the risk of PAC was significantly higher in pregnancies ending in an abortion as compared with those ending in a birth (OR = 1.22; 95% CI: 1.09–1.37) [4]. Furthermore, in Apulia, southern Italy, about 60% of pregnancies of women diagnosed with PAC ended in abortion, and the risk of PAC was higher in pregnancies resulting in miscarriage (OR = 1.26, *p*-value < 0.05) [3].

Our results are broadly consistent with those findings: the incidence rate (per 1000) of PAC was 1.29 for births and 1.52 for abortions. However, in the last two years analysed, the rate of PAC was higher for births than for abortions (1.42 vs 1.21 per 1000, respectively). The abortion rate observed in our analysis is markedly lower than Apulia; it is difficult to explain this difference, but Apulia is one of the Italian regions that report among the highest rates of induced abortion in the country [7, 8]. In addition, the analysis from Apulia covered a period partly prior to the one we considered, and the observed abortion rate tended to decrease over time.

This finding is consistent with the decrease in termination of pregnancies reported in a population-based study from Denmark [9]. Improvements in the management of pregnant cancer patients, including increased diagnosis of early indolent cancers and greater awareness of chemotherapy, may have contributed to the downward trend [10–15]. As a result, oncological therapy has been preferred to abortion, and the number of live births has increased over time [10, 16]. Knowledge of the feasibility and safety of oncological treatments during pregnancy has also increased, leading to more frequent pregnancy continuation during cancer treatment [12]. According to the International Network on Cancer, Infertility and Pregnancy, an increasing proportion of women are receiving oncological treatment during pregnancy, with iatrogenic premature births decreasing [9, 13].

The diagnosis of cancer during pregnancy is challenging due to the interaction between the oncological condition and the treatment required, the health of the mother, and the health of the fetus. Several factors can affect the outcome of pregnancy in women with cancer, particularly when the cancer is diagnosed in the early stages of pregnancy. This may lead to an increased risk of miscarriage. The type, anatomical location and stage of the tumour play a crucial role in the complications of pregnancy; some tumours can be particularly problematic posing greater challenges, while others may be easier to manage.

In addition, as for treatment, the teratogenicity of chemotherapeutic drugs heavily depends on the timing of exposure. The use of chemotherapy in the first trimester is associated with an increased risk of miscarriage, fetal death, and congenital malformations, as organogenesis occurs in the first trimester [17, 18]. The effect of delaying treatment on maternal survival needs also to be considered. In the subgroup of PAC diagnosed in the first months of pregnancy, the abortion was 2.5 times more frequent than the continuation of pregnancy, becoming about 4 times more frequent if only induced abortions were considered. Also, diagnoses in the first trimester after the pregnancy ended were more common in women who aborted in comparison with women who delivered; it is possible that part of those diagnoses was suspected during pregnancy and influenced the decision to terminate the pregnancy.

Regarding chemotherapy, in women who gave birth, treatment was started after pregnancy in about 90% of cases. Treatment was started during pregnancy in almost 40% of cases in women who aborted. This suggests that some abortion may be dependent on the start of treatment and that some cases may be more severe than those diagnosed in women who had given birth.

From a psychosocial point of view, the diagnosis of cancer has a significant impact on the couple’s decision to terminate the pregnancy. Mothers and couples face multiple stressors as they are forced to deal with the conflicting life events of the new life associated with pregnancy and the potential death due to cancer. A strong support system, including family, social relationships, and healthcare professionals, can help women cope with the challenges of pregnancy and cancer. From a clinical point of view, multidisciplinary and specialist case management can help make informed decisions about pregnancy, taking into account the specific oncological condition and treatment plan, also according to individual circumstances.

The strengths and limitations of this analysis should be considered. The NHS provides comprehensive coverage to all residents. Therefore, the administrative data used in this study cover the entire population and are not subject to selection bias. In addition, the HCU databases contain data that are consistently collected from many subjects over a long time period, allowing the exploration of temporal trends. A limitation of administrative data relates to the lack of clinical details which precludes a finer breakdown of cancer characteristics, treatments as well as long-term aspects of survival. Second, we defined cancer diagnosis as the first hospital admission due to a malignant disease. However, if the first diagnosis was performed in an outpatient setting, hospital data may not identify the exact time of diagnosis. Therefore, our estimates of PAC may be underestimated. Furthermore, we were unable to include all spontaneous abortions due to poor ascertainment in administrative data, especially in the case of early miscarriages that do not lead to hospitalisation. We were also not able to distinguish between voluntary induced abortions by maternal choice and induced abortions by therapeutic indication and have no information on the possible presence of malformations.

## Conclusion

In the current investigation, women with PAC had approximately 10% increased risk of abortion compared to cancer-free women. However, this association appeared to be decreasing in more recent time periods. This reduction in the association seems to be influenced more by spontaneous abortions than by induced abortions. Monitoring the trend in pregnancy outcomes in relation to PAC diagnoses is important and allows us to assess the results of improvements in the management of pregnant patients with cancer and to raise awareness among women and their doctors about the efficacy, safety, and feasibility of cancer treatment during pregnancy. From an epidemiological perspective, there is a need for further targeted studies with comprehensive clinical data to throughly analyse the specific determinants of abortion in women with PAC.

### Supplementary Information

**Additional file 1:** **Figure S1.** Flow-chart of selection of study cohort. Lombardy, Italy, 2010-2020. **Table S1. **Timing of diagnosis of pregnancy-associated cancer (PAC) among women who delivered and women who aborted. Lombardy, Italy, 2010-2020.  **Table S2.** Chemoterapy patterns according to pregnancy outcome and timing of treatment.  Lombardy, Italy, 2010-2020. 

## Data Availability

The data that support the findings of this study are available from Lombardy Region, but restrictions apply to the availability of these data, which were used under license for the current study. The data used in this study cannot be made available in the manuscript, the supplemental files or in a public repository due to Italian data protection laws. The anonymized datasets generated during and/or analyzed during the current study can be provided on reasonable request, from the corresponding author, after written approval by the Lombardy region.
