# Supplementary material for: Spontaneous and induced abortions in women with a diagnosis of gestational related neoplasm: a population-based linkage study in Lombardy, 2010–2020
Source: BMC Womens Health. 2023 Nov 8;23:586. doi: 10.1186/s12905-023-02685-6 (PMC10633990; doi:10.1186/s12905-023-02685-6)
Supplement: Supplementary file 1 — Additional file 1: Figure S1. Flow-chart of selection of study cohort. Lombardy, Italy, 2010-2020. Table S1. Timing of diagnosis of pregnancy-associated cancer (PAC) among women who delivered and women who aborted. Lombardy, Italy, 2010-2020. Table S2. Chemoterapy patterns according to pregnancy outcome and timing of treatment. Lombardy, Italy, 2010-2020. [file 12905_2023_2685_MOESM1_ESM.docx]

**
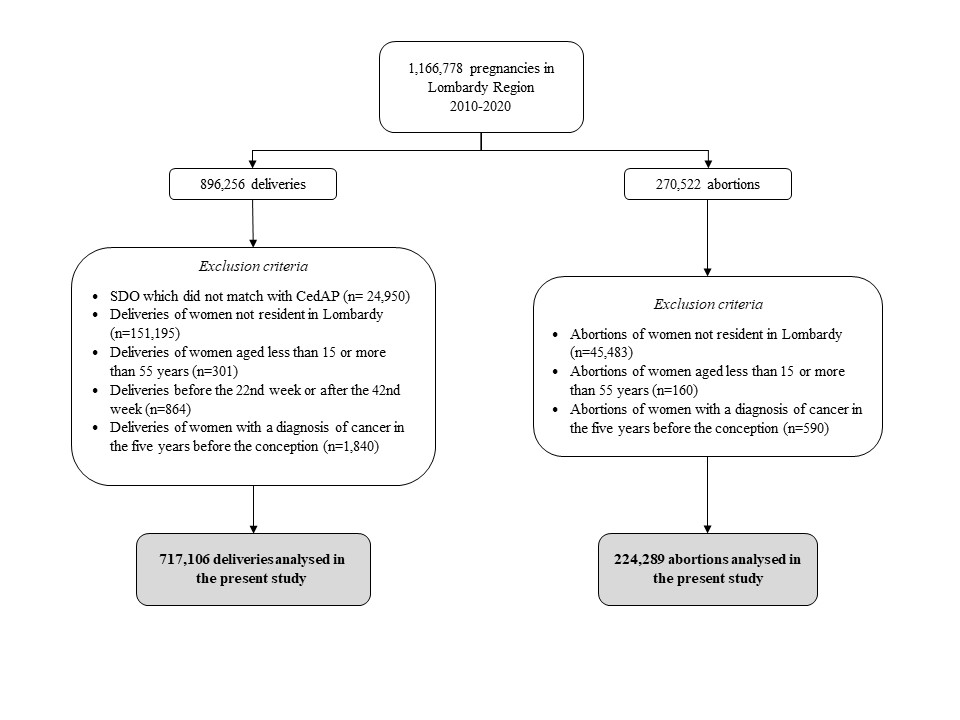
**

**Figure S1.**  Flow-chart of selection of study cohort. Lombardy, Italy, 2010-2020.

**Table S1.** Timing of diagnosis of pregnancy-associated cancer (PAC) among women who delivered and women who aborted. Lombardy, Italy, 2010-2020.

|  | **PAC** | |
| --- | --- | --- |
|  | **N** | **%** |
| *Births* |  |  |
| Diagnosis during gestation |  |  |
| First trimester | 21 | 11.6 |
| Second trimester | 50 | 27.6 |
| Third trimester | 110 | 60.8 |
| Diagnosis post-partum |  |  |
| 0-6 months | 329 | 44.2 |
| 7-12 months | 416 | 55.8 |
| *Abortions* |  |  |
| Diagnosis during gestation* | 34 | - |
| Diagnosis post-partum |  |  |
| 0-6 months | 168 | 54.7 |
| 7-12 months | 139 | 45.3 |

* Three months before the date of abortion.

**Table S2.** Chemoterapy patterns according to pregnancy outcome and timing of treatment. Lombardy, Italy, 2010-2020.

|  | **Births, N=407** | | **Abortions, N=155** | |
| --- | --- | --- | --- | --- |
| Timing of treatment | **n** | **%** | **n** | **%** |
| Only during pregnancy | 10 | 2.5 | 2 | 1.3 |
| Only during postpartum | 361 | 88.7 | 95 | 61.3 |
| During pregnancy and postpartum | 36 | 8.8 | 58 | 37.4 |
